# Stakeholder views regarding cultural diversity teaching outcomes: a qualitative study

**DOI:** 10.1186/1472-6920-5-37

**Published:** 2005-11-01

**Authors:** Nisha Dogra, Olivia Carter-Pokras

**Affiliations:** 1 University of Leicester Greenwood Institute of Child Health, Leicester, UK; 2 University of Maryland School of Medicine, Baltimore, MD, USA

## Abstract

**Background:**

Cultural diversity teaching is increasingly present in both undergraduate and postgraduate training programmes. This study explored the views of stakeholders in medical education about the potential outcomes of cultural diversity teaching and how they thought cultural diversity programmes might be effectively evaluated.

**Methods:**

A semi-structured interview was undertaken with 61 stakeholders (including policymakers, diversity teachers, students and users). The data were analysed and themes identified.

**Results:**

Many participants felt that clinical practice was improved through 'cultural diversity teaching' and this was mostly as a result of improved doctor-patient communication. There was a strong view that service users need to participate in the evaluation of outcomes of cultural diversity teaching.

**Conclusion:**

There is a general perception, rather than clear evidence, that cultural diversity teaching can have a positive effect on clinical practice. Cultural diversity teaching needs to be reviewed in undergraduate and postgraduate medicine and better evaluation tools need to be established.

## Background

Cultural diversity teaching has been advocated for over forty years in the US and more recently in the UK [1,2]. One of the major justifications for it has been that it will help reduce healthcare disparities. The Sainsbury Centre for Mental Health reported a case in which a young black man died after being inappropriately restrained. It accused the National Health Service of being racist, and advocated cultural awareness training for healthcare professionals [3]. This paper reports on stakeholders' views about the perceived outcomes of cultural diversity teaching. The discussion considers the implications for undergraduate and postgraduate medical education.

The use of 'cultural diversity' in this paper is broadly consistent with the definition of culture adopted by the Association of American Medical Colleges (AAMC) Task Force [4] in its report on *Spirituality, cultural issues and end of life care*. AAMC noted that:

"Culture is defined by each person in relationship to the group or groups with whom he or she identifies. An individual's cultural identity may be based on heritage as well as individual circumstances and personal choice. Cultural identity may be affected by such factors as race, ethnicity, age, language, country of origin, acculturation, sexual orientation, gender, socioeconomic status, religious/spiritual beliefs, physical abilities, occupation, among others. These factors may impact behaviours such as communication styles, diet preferences, health beliefs, family roles, lifestyle, rituals and decision-making processes. All of these beliefs and practices, in turn can influence how patients and heath care professionals perceive health and illness and how they interact with one another" [4].

One of the attitudinal objectives outlined in the first *Tomorrow's Doctors *[2] was that:

"At the end of the course of undergraduate medical education the student will have acquired and will demonstrate attitudes essential to the practice of medicine including respect for patients and colleagues that encompasses, without prejudice, diversity of background and opportunity, language, culture and way of life". [GMC, 1993: 15]

By publishing *Tomorrow's Doctors*, the GMC set the framework within which it expected medical education in the UK to develop. Although the GMC and the Quality Assurance Agency (QAA) regularly visit medical schools to monitor standards of medical education, medical schools remain free to develop curricula as they see fit. The drive behind *Tomorrow's Doctors *was a desire to overhaul medical school curricula. There was, in part, an acknowledgement of the growing evidence that curricula could not continue to expand at the same rate as medical knowledge [5-7]. As well as a reduction of emphasis on acquiring fact-based knowledge, *Tomorrow's Doctors *[2] placed increased emphasis on human skills and the ability to communicate with patients and colleagues. The GMC recognised changes in society and the need for changes towards improving equality [8]. This was maintained in the second edition [9].

Changes in undergraduate education have now been followed by the establishment of a Postgraduate Medical Education and Training Board (PMETB [10]) to regulate postgraduate education. Modernising Medical Careers (MMC [11]) is a relevant document in that it places similar expectations on postgraduate education as *Tomorrow's Doctors *did on undergraduate education. Although MMC does not explicitly mention diversity, the expectation is that learning outcomes will be set against the attributes in Good Medical Practice [12] to shape the structure for appraisal and revalidation of doctors. GMC clearly expects doctors to respect diversity and cultural diversity learning is seen as a lifelong objective..

### Evaluation of specific cultural diversity programmes

When considering the need for an evidence-based approach to medical education, it is clear that evaluation of new programmes needs attention. Few cultural diversity teaching programmes have been subject to evaluation beyond subjective student feedback [13,14]. There are exceptions, which used pre- and post-teaching questionnaires [15-20]. All reported some degree of short term 'positive' changes in student attitudes, but none of the authors assessed long-term change in student attitudes Evaluating the effectiveness of cultural diversity teaching is difficult so the issue of evaluation is perhaps sidestepped.

Webb and Sergison [21] reported that the participants on their cultural diversity program found the training useful and staff commented on how they thought their own behaviour had changed at follow-up. However, there were no objective measures of change. Examples of changes of practice included using more culturally appropriate pictures for the ward and not using minors as interpreters.

The U.S. Task Force for Preventive Services conducted a systematic review of five interventions to improve cultural competence in healthcare systems including cultural competency training for healthcare providers [13]. The Task Force identified only one study that they felt had a fair quality of execution, and therefore concluded that the evidence was insufficient to determine the effectiveness of cultural diversity training for healthcare providers. A more extensive review by the Agency for Healthcare Research and Quality found excellent evidence for improvement in provider knowledge, good evidence for improvement in provider attitudes and skills, and good evidence for improvement in patient satisfaction [14]. Both reviews found a lack of consistency in intervention methods and measured outcomes [13,14].

This report is part of a PhD thesis [22] on the views held by key stakeholders in medical education on the teaching and learning of cultural diversity. This paper reports on the findings which explored the views of stakeholders in medical education about the potential outcomes of cultural diversity teaching and how they thought cultural diversity programmes might be effectively evaluated.

## Method

### Devising the interview schedule

Semi-structured in-person or telephone interviews were conducted with mostly open-ended questions [23]. It was concluded some structure was necessary to answer the specific research questions relating to the understanding of cultural diversity, its teaching and assessment.

The interview schedule drew on the literature base in sociology, medical education, education and intercultural studies [22]; previous research [20]; clinical, educational and personal experience; earlier interviews with members of the General Medical Council (GMC) Education Committee responsible for the first *Tomorrow's Doctors *[2]; and an Internet search of all UK medical school websites.

The interview was conducted in three parts. Part I collected basic demographic data (age and gender), as well as roles and experience. Part II began with four open-ended questions and participants talked unprompted, with the interviewer clarifying if necessary. The four questions were:

• How do you understand the term "cultural diversity"?

• What do you think should be taught at undergraduate level about cultural diversity?

• What main topics do you think that cultural diversity teaching should encompass at undergraduate level?

• How do you think cultural diversity should be taught?

The interview then continued open-ended but focused on specific aspects of teaching such as learning outcomes, delivery methods, assessment, and influence on clinical practice and student perspectives. Part III asked for the ways in which participants used or understood key terms such as race, ethnicity and multiculturalism. The interview concluded by asking participants of their experience and/or training in cultural diversity.

The interview was piloted with two policymakers and one diversity teacher and minor modifications were then made to the schedule. A flexible design was used when delivering the interviews. Modifying the interviews was part of the research process [22]. The research topic and themes remained the same, but the exact wording was modified as the interviews progressed enabling more themes to be explored as necessary. For example, the question about how learning outcomes might be phrased, was not asked after the first three interviews because participants struggled to respond with appropriate objectives immediately. They knew what they wanted to cover but could not be specific in voicing them. As the exact wording is less important than the overall aim and purpose of the teaching, this question was omitted: persisting with it would have been time-consuming and would not have yielded useful data. It may also have been frustrating for participants. The interview schedule is included as an Appendix One.

### Sample and sample size

There were two stages of sampling to ensure a wide range of participants: selection of different groups of stakeholders followed by selection of individuals from these groups. Four UK medical schools from which staff could be interviewed were also identified for the study (see below). Selection was not random as key individuals were targeted. A minimum target of 50 interviews was set at the start of the study due to available time and resources, and to allow valid comparisons between different stakeholders. The sample group included:

• **Communication teachers **(n = 6): teachers responsible for communication skills training;

• **Curriculum heads **(n = 7): heads of medical education and curriculum committee members who implement policy;

• **Diversity teachers **(n = 7): teachers are responsible for developing and delivering cultural diversity;

• **Policymakers **(n = 18): members of organisations that decide or influence policy on medical education (e.g. General Medical Council);

• **Researchers **(n = 2): included researchers in 'cultural diversity' and associated areas actively teaching on ethnicity;

• **Students **(n = 7): medical students;

• **Users **(n = 7): included patients, patient representatives and advocates.

Table 1 details how medical schools included in the sample were identified.

**Table 1:** 'Cultural expertise' and 'cultural sensibility' [24]

***Cultural expertise***: Expertise is defined as expert skill, knowledge or judgement with an expert being defined as having special skill at a task or knowledge in a subject. There is a notion that through learning knowledge about 'other' cultures, one can develop *cultural expertise*. In learning terms this means learning about the *modus operandi *of different cultural groups.
***Cultural sensibility***: Sensibility is defined as openness to emotional impressions, susceptibility, and sensitiveness. It relates to a person's moral, emotional or aesthetic ideas or standards. If one is open to the outside, one might reflect and change because of that experience. There is no notion of acquiring expertise about others but a recognition that we need to be aware of our perspectives and how they affect our ability to have an openness about other perspectives. This leads to a generic openness to diversity of all kinds and specific knowledge is not taught.
On this basis the following types of medical schools were included: Where the cultural diversity programmes were more consistent with *'cultural sensibility '*than *'cultural expertise'*; Where the programmes had elements of *'cultural sensibility*' and elements of *'cultural expertise' *model; Where the programmes were more consistent with *'cultural expertise' *but had some aspects of *'cultural sensibility'; *and Where no specified programme regarding cultural diversity was taught (this was based on information available about the curriculum and direct contact with staff at the school).

The sampling strategy ensured that interviews continued until saturation was achieved. Using purposive sampling and 'snowballing', a total of 61 individuals were interviewed. With snowballing, individuals who have been interviewed are asked after their interviews to identify other members of their group who may usefully act as informants [17]. Formal association with a medical school was defined as being employed by the medical school (including clinical NHS staff appointed as honorary teachers and external examiners), or being a student at a UK medical school. Individuals from 14 of the 26 established medical schools in the UK were involved (two schools have campuses at two sites; therefore, 12 curricula were effectively covered). These 14 schools were not specifically selected although we did ensure that schools within the above categories were part of the overall sample. The schools are representative of the UK overall. Members from eleven policymaking organisations and six medical disciplines were interviewed. Other 'clinical' perspectives included pharmacy, social work, community youth work and nursing. Non-clinical participants came from sociology, anthropology, accountancy, research and advocacy work.

### Procedure

Interviews took place face-to-face as a first preference and by telephone, if the former was not possible. Initial contact was through a formal introductory letter that invited the participants to contact the researcher if there were any queries. The letter also stated that the interviews would be confidential and that the local research NHS ethics committee had approved the project. If there was no response, the initial letter was followed-up by email or by a second letter until the target number was achieved. No one was contacted more than twice if they failed to respond. Most participants replied by letter or email to agree to take part; copies of these correspondences were considered written consent to participate and kept. Twelve individuals responded but declined to participate for a variety of reasons including not being an appropriate person, too busy and no longer in post. Ten of these were policymakers and two were curriculum leads. A further 17 individuals did not respond; seven were user representatives or organisations, four policymakers, three were teachers, two researchers and one students. As we had recruited enough participants only one of these received a reminder.

Interviews were audiotaped and transcribed verbatim. Of the 61 participants, teachers and policymakers comprised the largest two groups, 45 were affiliated with medical schools, 39 were men, 31 were clinically active, 50 were White British, and 42 were between 41–60 years of age (see Table 2). At the outset, the researcher explained how the term 'cultural diversity' was being used in the study but interviewees were able to use the term as they thought appropriate.

**Table 2:** Summary demographics of participants

**Number**	**Ethnicity**	**Gender (M= male F = female)**	**Mean Age Range in years**	**Medical school connection**	**Currently in Clinical Practice**
**Communication teachers **(n = 6)	5 White British 1 mixed race	3 M 3 F	46–50	6	4
**Curriculum heads **(n = 7)	7 White British	4 M 3 F	46–50	7	5
**Diversity teachers **(n = 14)	13 White British 1 Indian	5 M 9 F	46–50	14	6
**Policymakers **(n = 18)	13 White 3 Indian 1 Bangladeshi 1 Pakistani	17 M 1 F	51–55	9	13
**Researchers **(n = 2)	2 White British	2 M	41–45	2	0
**Students **(n = 7)	6 White British 1 Indian	3 M 4 F	Under 30	7	In training
**Users **(n = 7)	4 White British 1 Black British 1 Indian 1 Pakistani	4 M 3 F	41–45	0	0
**TOTAL **(n = 61)	50 British 6 Indian 2 Pakistani 1 Bangladeshi 1 Black 1 Mixed Race	39 M 22 Female	46–50	45	28

### The researcher's part in the research and interview process

This research was undertaken by ND, a female, of Indian origin, aged forty, brought up and educated in the UK, who works as a senior clinical academic in child and adolescent psychiatry at an East Midlands medical school. Having undertaken the development of a module in 'cultural diversity', she had some professional familiarity and experience with the topic. As Robson [25] has highlighted all these factors may influence the research.

### Analysis

The analysis in this study was a combination of the quasi-statistical and template qualitative methodology [25] and followed a series of systematic steps. Responses were also counted to enable comparison between groups [26]. The content of the interviews was analysed manually to identify word and phrase frequencies and inter-correlations. Key themes were identified from the texts as a whole and from collations of responses to specific themes. Key themes were categorised under origins, organisation, contents, delivery and outcomes of the curriculum. The justification of this was the need to relate the findings to existing theory. The process of analysis for this research study took into account the steps outlined by Miles and Huberman [27]. The themes were organised under the major topics of origins and development, organisation, contents and delivery; and outcomes.

## Results

Although this was essentially a qualitative study, some quantitative data are inevitably presented to illustrate the spread of responses. These are included in the text as appropriate and summarised in Tables 3, 4, 5, 6. Direct quotes are presented in the findings to illustrate points made. Quotes are also integrated into the discussion to highlight themes identified through qualitative analysis. Where no difference between the different stakeholders' perspectives is mentioned, the views were found across the range of stakeholders. In general, the findings showed no discernable pattern between sections of the sample, although students and service users were most optimistic about the outcomes of cultural diversity teaching.

**Table 3:** Does 'cultural diversity' teaching affect practice?

	**Improved Practice**	**Made a Difference**	**Did Not Know**	**Unsure**	**Too Early to Say**
**Communication Teachers **(n = 6)	2	1	2	1	
**Curricular Heads **(n = 7)	3	2	1	1	
**Diversity Teachers **(n = 14)	9	1	3		1
**Policymakers **(n = 18)	9	4	3	2	
**Researchers **(n = 2)	1	1			
**Students **(n = 7)	6				
**Users **(n = 7)	5	1		1	1
**TOTAL **(n = 61)	27	10	9	5	2

**Table 4:** How might 'cultural diversity' teaching affect practice?

	**Improve Doctor/Patient communication**	**Improve Student Awareness**	**Improve Patient Satisfaction**	**Improve Sensitivity to Patients**	**Better Health Care**	**Better Access to Care**
**Communication Teachers **(n = 6)	2	1				
**Curricular Heads **(n = 7)	2	2		1		
**Diversity Teachers **(n = 14)	5	6			1	
**Policymakers **(n = 18)	12	4			1	1
**Researchers **(n = 2)		1		1		
**Students **(n = 7)	2	2		1		
**Users **(n = 7)	4			2		
**TOTAL **(n = 61)	27	16	5	4	2	1

**Table 5:** How do you think programmes that endeavour to teach cultural diversity might be evaluated?

	**Observe Students in Practice**	**Assessment**	**Longer-term evaluations**	**Relate to Learning Outcomes**	**Student evaluation**	**Ask Users**	**Unsure – Probably Difficult**	**Research Needed**	**Ask Employers**	**Reflective Portfolios**	**Focus Groups**
**Communication Teachers **(n = 6)		1	3			1					1
**Curricular Heads **(n = 7)	4	1		4					1		
**Diversity Teachers **(n = 14)	5	4	3	1	1	1		2	1	1	
**Policymakers **(n = 18)	3	3	1	3	2	1	2	1			
**Researchers **(n = 2)	2									1	
**Students **(n = 7)	1		1		1	1	1				
**Users **(n = 7)					1						
**TOTAL **(n = 61)	15	9	8	8	5	4	3	3	2	2	1

**Table 6:** How might the impact on practice be measured?

	**User Involvement**	**Observed in Practice**	**Long Term Outcomes**	**Research Needed**
**Communication Teachers **(n = 6)	4		1	
**Curricular Heads **(n = 7)	2	1	1	
**Diversity Teachers **(n = 14)	8	4	2	3
**Policymakers **(n = 18)	8	3		2
**Researchers **(n = 2)				1
**Students **(n = 7)	6	1		
**Users **(n = 7)	4			
**TOTAL **(n = 61)	32	9	4	6

Other responses were: ask employers (3); unsure (3); self-refection (3); complaints register (3); OSCES (2); ask students if felt prepared (2); patient care improves (1); audit (1); improved compliance (1) and comparing services with the National Service Framework (1).

The key questions about the outcomes were:

• Does cultural diversity teaching make a difference?

• How does it make a difference?

• How might programmes teaching cultural diversity be effectively evaluated?

### Does cultural diversity teaching make a difference?

The majority of participants (35) felt that clinical practice was improved through cultural diversity teaching and that this was mostly through improved doctor-patient communication. The outcomes in clinical practice were related to a process as opposed to better knowledge with which to serve patients. This is important as this highlighted inconsistencies between what participants thought should be taught and what the anticipated outcomes were.

Only five participants stated that patient satisfaction might be improved through patients feeling better heard, valued and understood and another five thought there might be improved sensitivity to the patient's concerns and values. Others did not comment as to how cultural diversity teaching might improve the doctor-patient relationship; that is whether it was through improved knowledge of the patient's background or through a collaborative partnership with the patient. Students and users were more confident than other participants that cultural diversity teaching did improve clinical practice.

Despite the lack of evidence of any clear effect on clinical practice [13,14], there are many advocates for the inclusion of cultural diversity in the curriculum, which is contrary to an evidence-based approach. The sample interviewed was more likely to be positive about cultural diversity as most of them had involvement or responsibility in this area. If there is a belief that cultural factors affect the way health is perceived in general, then the logical step is to believe that learning about the subject is important. The work is undermined if any doubt is expressed regarding its effectiveness. Politically it may be unacceptable to say there is very little evidence that the teaching of cultural diversity makes any difference, especially if organisations have invested money on its teaching. However, the views that it does make a difference are made in the absence of firm evidence to support this perspective although there is also no clear evidence to indicate that training is in any way detrimental.

When asked if participants thought cultural diversity teaching made a difference to clinical practice, no one thought it did not. Thirty-five participants felt that it improved practice with two of these saying only if it was taught properly. Another thought it might, but had some reservations. Another felt that success was dependent on the ethos of the organisation.

*"It is inevitably bound to have, both in terms of how you relate to colleagues as well as how you relate to patients. I think if it is well done it spins off beyond the obvious interface. You get to start seeing people as individuals, not as white or black, or whatever else, but actually as real people and not classify them as Catholics or Irish or whatever. Yes, I think it changes your perspective" *(R30: Policymaker)

One participant gave an affirmative response based on her experience of delivering a postgraduate programme.

*"I can only talk about our pack because that's the only one I have got experience and have evaluated. People reported quite a lot of things that they changed as a result of being on that. For example a lot of people said that they would find an interpreter, whereas before they might have muddled through... A couple of other people said they felt confident to challenge racism when they saw it. Someone else said he or she had changed the language they used to describe black clients, which was quite interesting. I think if you do win people over all kinds of things can happen really" *(R27: Diversity teacher)

This suggests minor behavioural changes may have significant clinical implications even without any change in attitudes. Ten participants hoped it made a difference, nine did not know and five were unsure if teaching cultural diversity made any difference. One diversity teacher and one student thought it was too early to say if teaching made a difference.

### How might cultural diversity affect clinical practice?

Participants were also asked how cultural diversity teaching might affect practice.

In terms of how cultural diversity teaching could affect practice, 51 gave a single response, 6 gave several responses in which cultural diversity teaching might make an impact and 4 were unsure. The number of responses is noted in parentheses below. Responses made included:

• Improved doctor/patient communication (27)

*"Well I would hope that it would mean that patients would feel that they were valued, that they were listened to, and that they were understood. Hopefully when those things happen patients respond better to whatever advice or however they are treated... I think it would have a huge impact on clinical practice" *(R5: Communication teacher)

• Student awareness would be improved about issues related to patients with one stating that this would lead to better communication (16)

*"Yes, by making students aware. There is absolutely no doubt about that. You can see from the realisation in student's eyes when you tell them something about a particular cultural or ethnic group" *(R20: Diversity teacher)

### How might cultural diversity programmes be evaluated?

The variety of responses and the lack of clarity of most of the responses to methods for evaluation of cultural diversity teaching programmes indicate that more work needs to be undertaken. Interviewees were asked how cultural teaching programmes might be evaluated and how the impact of cultural diversity teaching on practice might be measured. Participants made the following responses:

• Thought that students needed to be observed in practice to effectively evaluate whether learning outcomes had been successfully met (15).

*"Ultimately they have got to be evaluated by whether you get the outcomes that were specified, but of course those outcomes are expressed in practice... I would suspect that the main focus would have to be on senior SHO type of positions where people have had a chance to reflect, and try to get at whether there has been an actual change. Of course you can't do any of the things you want to do; you can't do any trials, or anything. It's very difficult to try and identify groups who have not been exposed to intervention or randomising in any way, but that's what you would be looking for... The difficulty here is that senior SHO level is quite a late point at which to offer remedial help" *(R10: Curriculum head)

*"They could be evaluated by people from culturally diverse backgrounds evaluating them as well. I think it would be important to have their input into the development, rather than just the evaluation" *(R11: Curriculum head)

This statement implies that because patients come from the non-majority they are able to effectively decide whether care is appropriate for a range of people. It does not acknowledge that patients may bring their own biases and prejudices.

*"I think that measuring of outcomes is so difficult when all the starting points are going to be different so I think we have to be looking at it from the perspective of portfolio learning and building up a portfolio. It's a very problematic area and one that needs to be examined more" *(R26: Diversity teacher)

This reflects a need for outcomes to be measured in several ways and then triangulated to see whether the initial outcomes have been met.

When asked how the impact on practice might be measured, 32 participants thought that user involvement was important. This might be through the use of questionnaires, focus groups, or in-depth interviews. One participant felt it important that user input be contextualised, and that we needed to be careful, as there may be a difference between what patients and doctors regard as effective. Nine participants thought that students should be observed in practice and a further two linked this to the process of revalidation. Four participants felt that longer-term outcomes were needed, and six felt that effective research was needed. Other responses were: health outcomes but none were specified (4); ask employers (3); unsure (3); self-refection (3); complaints register (3); Objective Structured Clinical Examinations (OSCES) (2); ask students if they felt prepared for diversity issues in practice (2); patient care improves (1); audit (1); improved compliance (1) and comparing services with the National Service Framework (1).

One participant linked undergraduate teaching with the pre-registration house officer years and made suggestions on the effectiveness of teaching in practice, but also noted that consultants willing to learn could present excellent role modelling opportunities.

*"You could say, 'right, regarding Doctor X, how do you think they perform in these particular aspects', so that you would actually have 360° assessment of every single member of the team by everybody else. You would have multiple observations, so that everybody would be feeding back about me to somebody else, albeit anonymously." *(R36: Policymaker)

The response below indicates some consistency with an evidence-based approach, and suggests that there is a need for different teaching approaches to be implemented and compared.

*"Ideally to have trial evidence for any teaching that we do, but that's obviously difficult. We can have comparative trials, non-randomised, with one half a year taking a particular model and the other group taking the Model B, and seeing what the outcomes were" *(R17: Diversity teacher)

The key findings were that over half the participants felt that cultural diversity teaching had a positive influence on clinical practice and that this was mostly through improved doctor-patient communication and student awareness. Just over half of the participants felt it was necessary to involve users in deciding whether practice was improved. Several different types of evaluation were suggested but there was a lack of clarity about this.

## Discussion

Many of those interviewed had a specific interest in cultural diversity teaching. It is perhaps unsurprising that despite the lack of evidence of any clear effect on clinical practice [5], they felt that cultural diversity should be included in the curriculum. However, there was also consensus that programmes had not been effectively evaluated. The implication here is that unless such teaching is shown to be detrimental, it must be a good thing but we need to be clear about what we teach and how we evaluate it.

The responses from this study indicate that a multifaceted approach to evaluation may be the most appropriate way forward; that is a range of different approaches. Comprehensive evaluation could involve the following:

1. Subjective student feedback on the usefulness and relevance of the teaching program

2. Objective measures of changes in student behaviour and attitudes using survey methods

3. User feedback

4. Staff perspectives of whether students had changed (clinical and non-clinical staff).

Each of these features of evaluation was identified by the study sample. There was a view that none of these on their own would be sufficient to demonstrate the effectiveness of a programme. Such an approach would enable triangulation of the different perspectives. Changes in student practice may be identified by observing students in clinical contexts (videotape, patient feedback, observer feedback), and noting whether they acknowledge the possibility that differences between themselves and the patients (e.g., ethnicity, gender, socioeconomic status, language) could influence the consultation. Their method of addressing this potential difficulty could also be assessed. Another format for evaluating changes in student practice may be the discussion of cases in peer groups. Two students may discuss a case while a third student observes their conversation. Observing students will be asked to note whether the students discussing the case made assumptions about the patient or disregarded the patient's perspectives. In this way, students may be supported in questioning their own and their peers' practice on a day-to-day level, thus making the issue of diversity an integral part of the clinical context.

Clinical staff could be asked to make specific observations about students who are placed with them and to comment in general terms on whether they feel students are aware of how to manage differences between themselves and patients in a way that optimises management options. However, approaches depend on the staff's level of training. Tang et al [28] found that students are often better equipped and more willing to manage diversity than their senior colleagues. However, this type of evaluation requires commitment and resources and a more rigorous research base, which has yet to be developed. In addition, the subject has little credibility when clinicians themselves have not been trained and so cannot support students once they are in clinical practice. The ambivalence that clinicians may express about cultural diversity training [28,29] may be related to the lack of a strong evidence base. It may also relate either to the fact that clinicians may not receive cultural diversity training or perceive the training they received as poor. Either way there is little evidence of a positive effect on clinical practice.

Long-term follow-up of students has to date been lacking and was commented on by some participants. This would not only allow educators to assess whether learning outcomes are met, but would also show how learning outcomes are applied in practice. However, this approach is not always practical and would again be time-consuming. There is though a need to develop an evidence base in this field.

To assist medical schools in the United States with the development, implementation and assessment of cultural competence education programs, the Association of American Medical Colleges, supported by a grant from the Commonwealth Fund, has convened experts to develop a Tool for the Assessment of Cultural Competence Training [30]. Principles and recommended standards for cultural competence education for healthcare professionals have also been developed through a consensus process funded by the California Endowment [31].

### Limitations of the study

It is important to acknowledge the constraints of trying to select the 'right' people to meet the research objectives. It may be that some staff not involved in 'cultural diversity' but involved in medical education, who do not value diversity, were unlikely to be identified or participate. Given that interviews rely on the relationship established between the researcher and the participants, there is always the limitation that the research can be contaminated by the characteristics of the researcher. In this project the researcher could be viewed by some as an insider as she was both a teacher and doctor. In contrast, other interviewees, such as students and users who were perhaps the least empowered of the groups interviewed, may have perceived her as an outsider. As the policymaker group is perhaps the most influential and fairly broad, more members of this group were invited to participate than from other groups. It is possible that their views are overrepresented. However, the range of views they cover is not dissimilar to the range of views of other groups.

The interview was an effective research tool, but it is possible that it may have been too structured for some participants. The clear focus on education may also have deterred non-educationalists from expressing their thoughts in case they were perceived to be 'wrong' or 'politically incorrect'. A broader sample may have provided a more representative picture of what happens within organisations, especially medical schools. This might have revealed the rivalries existing between different subject teachers in an ever-expanding curriculum. The interview data might have been usefully triangulated with a questionnaire survey for additional breadth and depth. Whilst the data may have been limited more perspectives might have been explored. The participants were offered confidentiality and assured that the responses would be presented in such a way that no link could be made to any individual or organisation. Despite, this assurance, the lack of opportunity for completely anonymous comment may have meant that participants only gave what they perceived to be acceptable responses.

## Conclusion

There is a general perception rather than clear evidence that cultural diversity teaching can have a positive effect on clinical practice. This is probably appropriate given the lack of strong evidence that currently exists about the effectiveness of such teaching. There is an urgent need to develop effective tools by which the effects of teaching on clinical practice can be measured including follow up of participants into their clinical practice. There is also a need to critically review cultural diversity programmes and question whether they are delivering what they set out to do. There is a great opportunity to consider approaches across disciplines and devise strategies to improve such education and the effect it has.

## Competing interests

The author(s) declare that they have no competing interests.

## Authors' contributions

ND conducted the study and analysed the data. OCP contributed to the writing of the paper.

## Appendix one: Summary of interview schedule

### I would like to discuss your personal views or views in your role regarding what you think should happen about teaching "Cultural diversity" to medical students

1. How do you understand the term "cultural diversity"?

2. What do you think should be taught at undergraduate level about cultural diversity?

3. What main topics do you think that cultural diversity teaching should encompass at undergraduate level?

4. How do you think cultural diversity should be taught?

5. Other areas of human diversity – from your perspective where do they fit in?

6. Should they be taught with cultural diversity or should cultural diversity be a separate course?

### I am now going to focus on specific questions about teaching of cultural diversity (seek justifications for response)

7. At which stage of the medical student's career should this teaching take place?

8. How much time do you think needs to be spent in this area?

9. What kinds of learning outcomes would you like to see established for this area?

10. What teaching strategies might be usefully employed?

11. Who do you think should teach cultural diversity?

### I would now like to move on to student assessment and student perspectives

12. Should students be assessed about cultural diversity?

13. How might they be assessed?

14. Should student feedback be gathered?

a. If so how might this be done?

b. How might student feedback be effectively used?

c. What might be your perspective if students said that they did not feel this kind of teaching was necessary?

15.

a. Would it be helpful to have guidelines on what should be taught?

b. What form might these take and who might develop them?

### I would now like to move on to specific programmes you may be aware of

16. What specific training programmes to teach cultural diversity are you aware of?

17. In your view, could these form models of best practice. (Prompt: have they used an evidence-based approach/been subject to critical evaluation?

18. In your opinion, how do you think programmes that endeavour to teach cultural diversity might be evaluated?

19. In your opinion, how might their impact on clinical practice be measured?

20.

a. Are you aware of the GMC perspective on this issue? If so, what is your understanding of it?

b. What is your perspective on the GMC including this issue in Tomorrow's Doctors?

21. In your view, does the teaching of cultural diversity have an impact on clinical practice?

22. If no, can you think of reasons why this might be the case?

23. If yes, can you think of how it impacts on practice?

### PART III

#### I would now like to move on to ask you your understanding of some key terms in this area. I should say that there is no right or wrong answer as such. I am just interested in your views?

What is your understanding of the following terms?

24. Culture

25. Ethnicity

26. Multiculturalism

27. Race

28. Cultural diversity

29. How do you think that the way that these terms are used and understood might influence medical education?

30. Do you have any personal training/experience in cultural diversity issues?

31. How would you classify your own ethnicity?

32. Is there anything else that you would like to add – either more about what we have covered or anything you feel I may have left out?

33. Finally, is there anyone else you think it would be useful for me to meet?

Thank you very much for you help with this project.

## Pre-publication history

The pre-publication history for this paper can be accessed here:


